# Supplementary material for: Regulation of protein abundance in genetically diverse mouse populations
Source: Cell Genom. 2021 Aug 31;1(1):100003. doi: 10.1016/j.xgen.2021.100003 (PMC9536773; doi:10.1016/j.xgen.2021.100003)
Supplement: Document S1. Figures S1–S8 [file mmc1.pdf]

Cell Genomics, Volume *1*

Supplemental information

Regulation of protein abundance in genetically diverse mouse populations

Gregory R. Keele, Tian Zhang, Duy T. Pham, Matthew Vincent, Timothy A. Bell, Pablo Hock, Ginger D. Shaw, Joao A. Paulo, Steven C. Munger, Fernando Pardo-Manuel de Villena, Martin T. Ferris, Steven P. Gygi, and Gary A. Churchill

Supplemental Figures

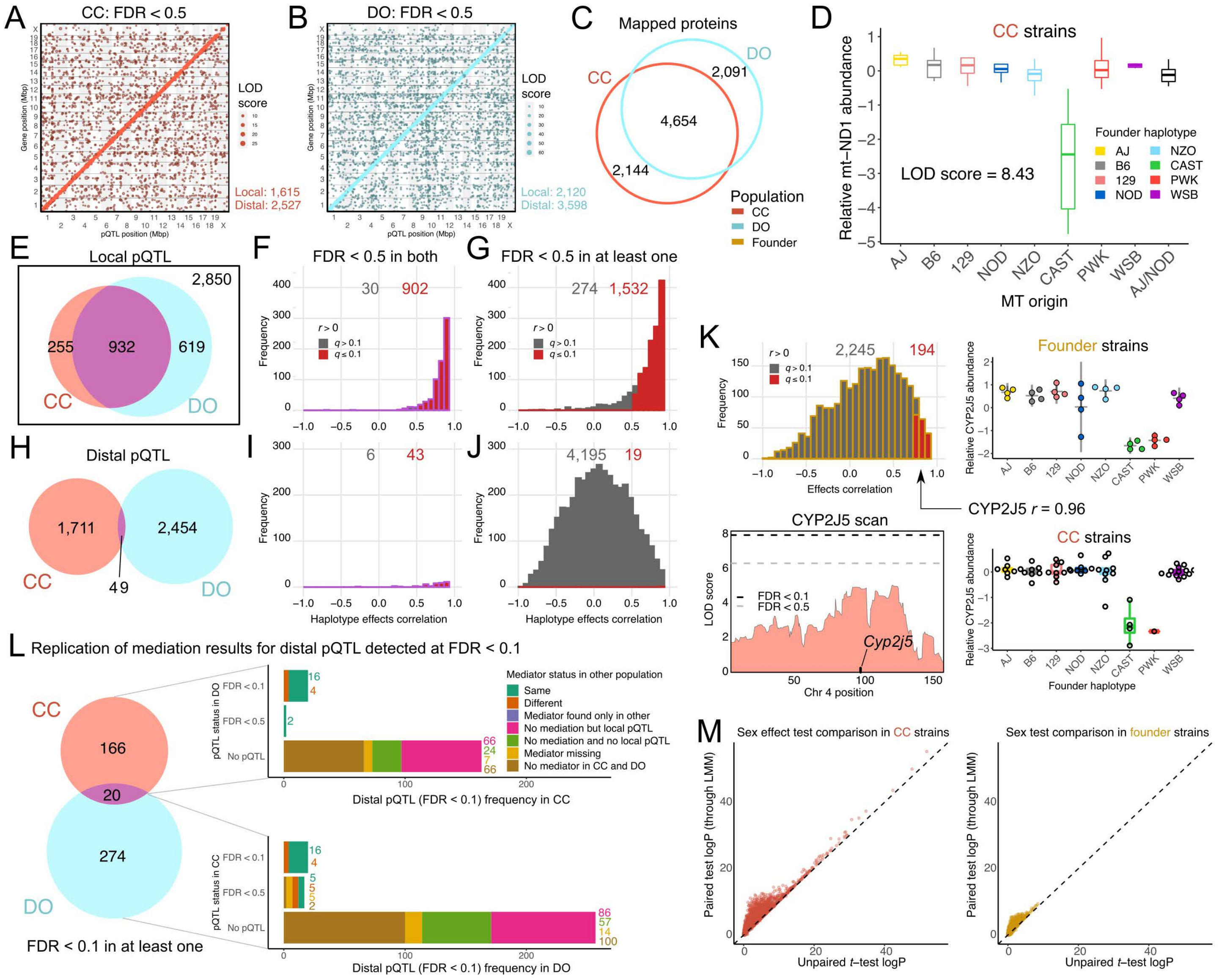

Figure S1. Comparison of lenient mapping results, mediation candidates, and sex effects among genetically diverse mouse populations, related to Figures 1 and 2. Leniently detected (FDR < 0.5) pQTL in the (A) CC and (B) DO. The pQTL are plotted by the genomic position of protein coding genes against pQTL position. Dot size is proportional to association strength (LOD score). (C) Venn diagram of analyzed proteins between the CC and DO. (D) Abundance for mt-ND1 in the CC mice**.** Mitochondrial inheritance was ambiguous for six strains (black boxplot). (E) Venn diagram of local pQTL detected in the CC and DO. (F) The correlation of haplotype effects for local pQTL detected in both the CC and DO. (G) The correlation of haplotype effects for local pQTL detected in at least one of the CC or DO. Red bars represent pQTL with significantly correlated effects (FDR < 0.1). (H) Venn diagram of distal pQTL detected in the CC and DO. (I) The correlation of haplotype effects for distal pQTL detected in both the CC and DO. (J) The correlation of haplotype effects for distal pQTL detected in at least one of the CC or DO. (K) The founder strains can reveal under-powered local pQTL in the CC (and DO). The correlations between strain effects in mice from the founder strains and haplotype effects at putative local pQTL for genes with rare founder haplotypes in the CC that did not map a local pQTL (FDR < 0.5) (top left). CYP2J5 had significant correlation (r = 0.96) between the strain effects in the founder strains (top right) with the local haplotype effects in the CC (bottom right), characterized by low CAST and PWK effects. Mean $\pm$ 2 standard deviation bars are shown for the founder strains. The CAST and PWK haplotypes were rare, resulting in reduced power to detect the *Cyp2j5* local pQTL (bottom left). Stringent and lenient significance thresholds are included as horizontal black and gray lines, respectively. (L) Comparison of mediation results for distal pQTL stringently detected (FDR < 0.1) in at least one of the populations. Color indicates how the top candidate mediators compared between the CC and DO. (M) Observance of both sexes within the (left) CC and (right) founder strains improves power to detect differences between the sexes (sex effects). Comparison of -$\log_{10}$(p-value), or logP, from a linear mixed effect model (LMM) accounting for strain replicates and an unpaired t-test. Identity lines included for reference. See also Tables S1-5.

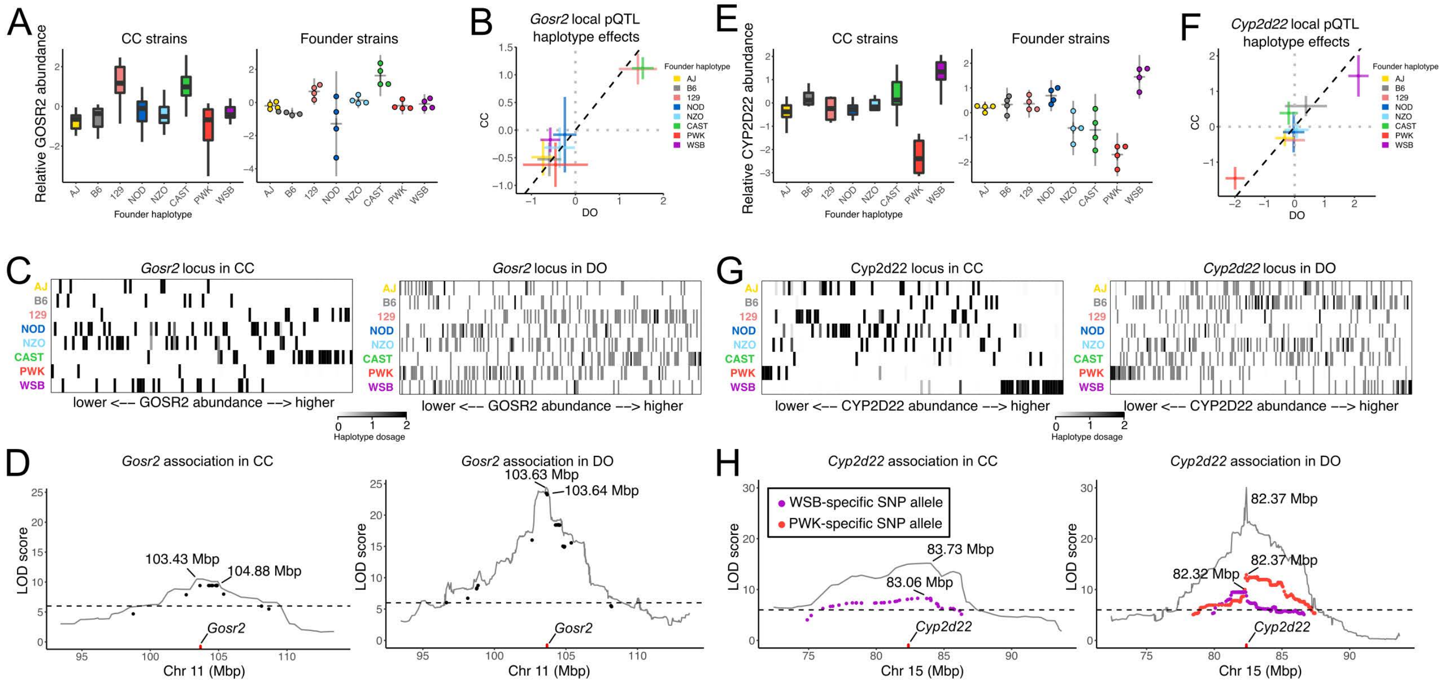

Figure S2. Examples of highly consistent local pQTL effects across the CC, DO, and founder strains, related to Figure 2. Local pQTL for (A-D) *Gosr2* and (E-H) *Cyp2d22* have highly consistent effects across the CC and founder strains. Mean $\pm$ 2 standard deviation bars are shown for the founder strains. The local pQTL effects are also highly consistent between the CC and DO, both in terms of modeled effects and actual data – for (B, C) *Gosr2* and (F, G) *Cyp2d22*. For comparisons of modeled effects (B, F), intervals represent standard error bars. Identity lines included for reference. To visualize the effects in actual data (C, G), the CC (left) and DO (right) data at the pQTL are represented as heatmaps with rows indicating founder haplotype dosages (expected counts) at the local pQTL and columns indicating individual mice, ordered by protein abundance. Clusters in rows towards the left or right sides represent founder haplotype effects, such as the high WSB effect on CYP2D22. Haplotype-based association scans at the pQTL are overlayed with variant associations for (D) *Gosr2* and (H) *Cyp2d22*. When the pQTL effects are approximately bi-allelic, as with *Gosr2* (D), the peak variant association and haplotype-based association are very close, consistent with a single variant driving the pQTL. SNPs with an allele shared by the 129 and CAST founder strains, matching the effects pattern, are shown. When the effects were more complex than bi-allelic, as with *Cyp2d22* (H), there are likely multiple causal variants present, and haplotype-based association produces higher LOD scores. WSB- and PWK-private variants with LOD score > 6 are included, highlighting linkage disequilibrium (LD) blocks that potentially carry founder-specific variants driving the extreme effects. The larger sample size and finer mapping resolution of the DO sample are evident in the higher LOD scores and narrower association peaks. Genomic positions of peak associations from variant- and haplotype-based mapping are marked. Horizontal lines at LOD score of 6 included as reference point across genome scans. See also Tables S4-5.

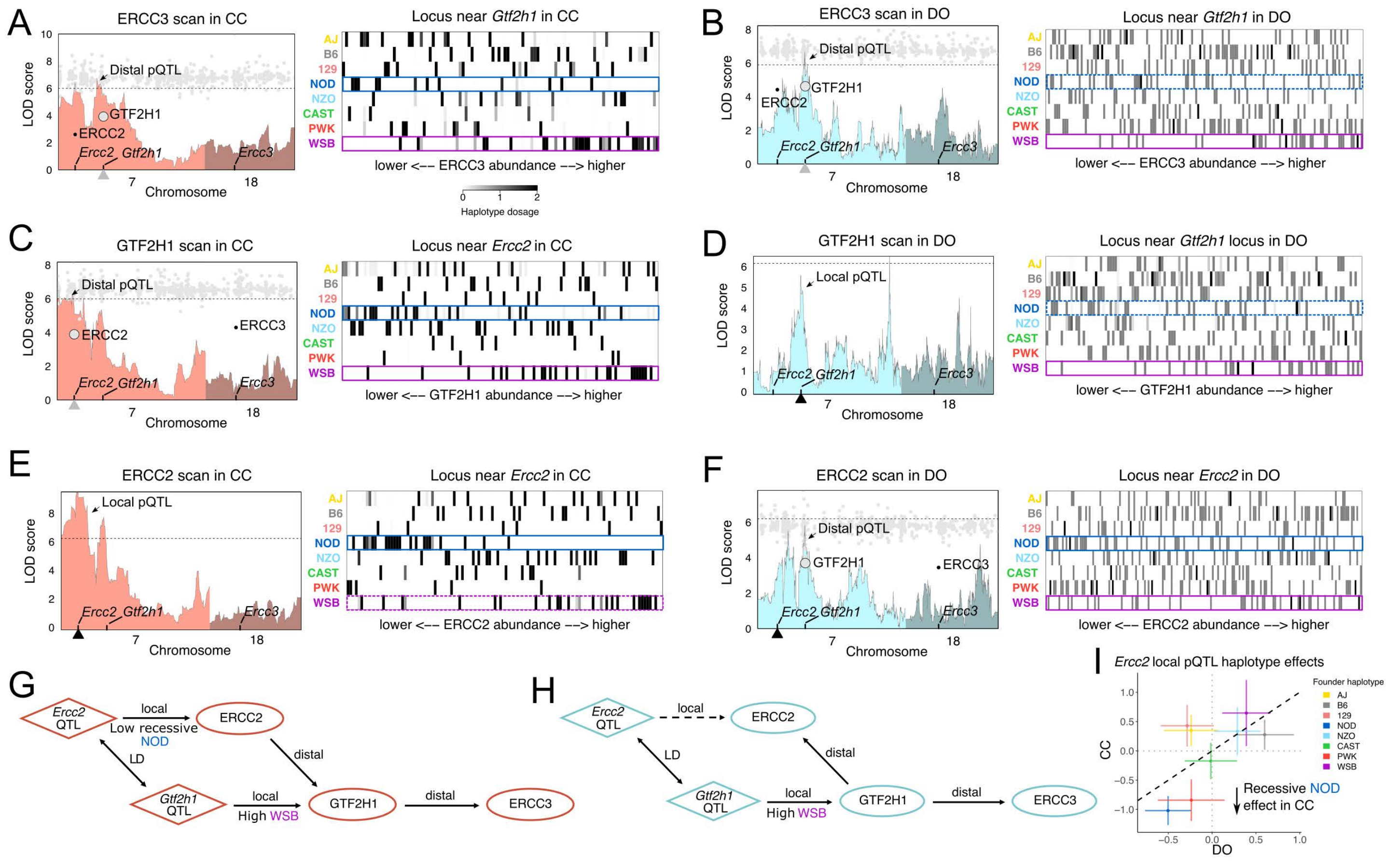

Figure S3. Similarity and differences in the genetic effects on ERCC3 between the CC and DO, related to Figure 2. Suggestive distal pQTL for *Ercc3* map to chromosome 7 in both the (A) CC and (B) DO, consisting of two peaks above *Ercc2* and *Gtf2h1*, genes that are approximately 30 Mbp apart and encode proteins known to interact with ERCC3. Mediation analysis identified GTF2H1 and ERCC2 as candidate mediators of the *Ercc3* pQTL. Gray dots represent mediation conditional LOD scores for proteins on chromosomes 7 and 18. Horizontal lines at LOD score of 6 included as reference point across genome scans. To better understand the pQTL effects, the founder haplotype inheritance was plotted as heatmaps with founder allele dosages (expected counts) as rows and individual mice as columns, ordered by ERCC3 abundance, at the chromosome 7 locus near *Gtf2h1*. A low NOD effect was observed, indicated by the dark blue box, which was weak to non-existent in the DO (dotted dark blue box), as well as a high WSB effect in both the CC and DO (purple boxes). (C) In the CC, *Gtf2h1* has a weak distal pQTL that mapped nearby *Ercc2* and was mediated by ERCC2 with similar low NOD and high WSB effects. (D) In the DO, *Gtf2h1* has a suggestive local pQTL (LOD score < 6) and notably no suggestive association near *Ercc2*. (E) *Ercc2* has a strong local pQTL in the CC (FDR < 0.1), driven by a low NOD effect, whereas in the (F) DO, a suggestive distal pQTL is observed near *Gtf2h1*. Diagrams for the relationships defined by pQTL and mediation in the (G) CC and (H) DO. In both populations, ERCC2 and/or GTF2H1 mediate suggestive *Ercc3* distal pQTL, and potentially affect each other as well. Teasing apart the directionality of effects is further complicated by the linkage disequilibrium (LD) between *Ercc2* and *Gtf2h1*. The low NOD effect at the *Ercc2* locus is stronger in the CC, and the high WSB effect is strongest at the *Gtf2h1* locus. (I) The haplotype effects at the locus near *Ercc2* are similar, though the NOD effect is more extreme in the inbred CC background. Standard error bars are shown. Identity line included for reference. See also Tables S4-5.

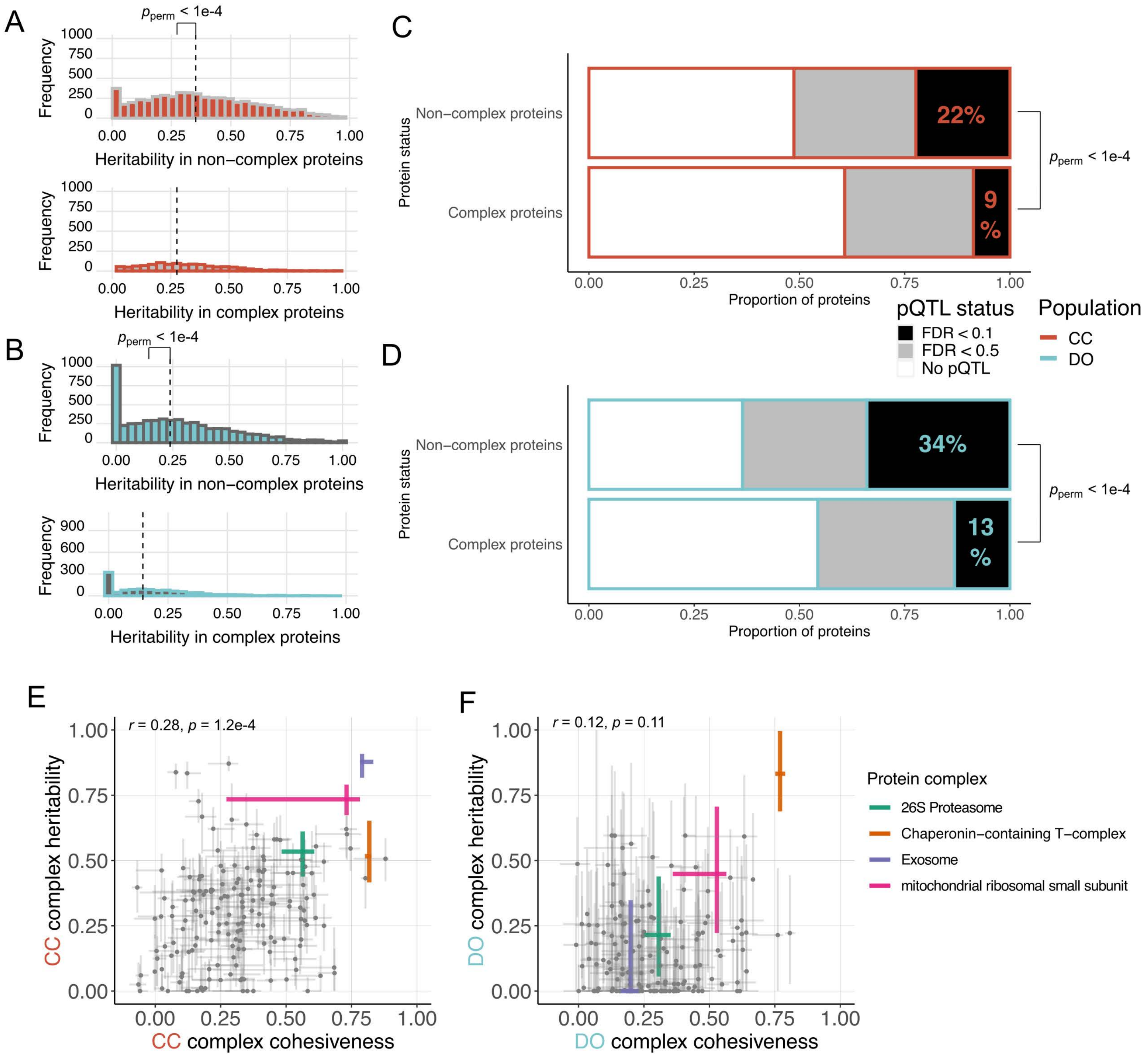

Figure S4. Reduction in detectable genetic effects on protein complex members, and the correlation between protein complex heritability and cohesiveness, related to Figure 3. Histograms of the heritability of abundance for proteins that are not members of protein complexes (top) and that are members (bottom) in the (A) CC and (B) DO. Vertical lines represent the median heritability, which significantly differed based on permuting complex member status (p_{perm} < 1e-4). A lower proportion of members of protein complexes map pQTL (p_{perm} < 1e-4) than non-members in both the (C) CC and (D) DO. The correlation between protein complex heritability and cohesiveness is stronger in the (E) CC (r = 0.33, p = 4.37e-6) than the (F) DO (r = 0.17, p = 0.03). Complex cohesiveness is represented as medians and interquartile ranges. Complex heritability was estimated from the first principal component (PC1) of the complex members, and bars represent 95% subsample intervals (Methods) around the estimate. Exosome, chaperonin-containing T-complex, 26S Proteasome, and the mitochondrial ribosomal small subunit are highlighted and are examined in greater detail (Figures 4-6, S5-7). See also Table S6.

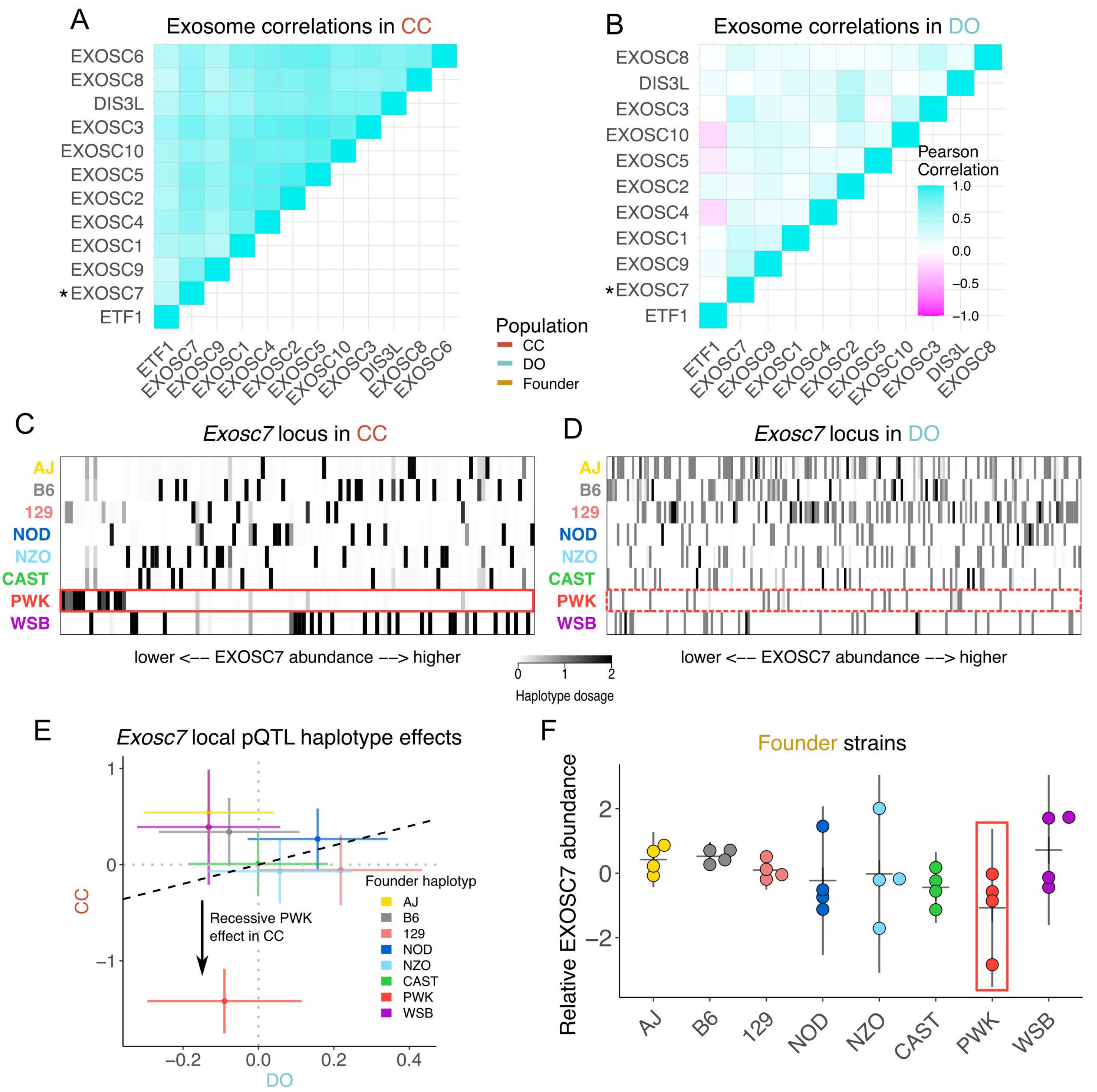

Figure S5. Genetic effects on the exosome complex are consistent with recessive mode of action through EXOSC7, related to Figures 3 and 4. Correlation of the exosome proteins in the (A) CC and in the (B) DO. The founder haplotype inheritance at the *Exosc7* local pQTL represented as a heatmap with founder haplotype dosages (expected counts) as rows and individuals as the columns, ordered by EXOSC7 abundance, for the (C) CC and (D) DO. The cluster on the left side in the PWK row (red box) for the CC reflects the low EXOSC7 abundance observed in CC mice that are homozygous for the PWK haplotype at the locus. The DO sample did not include any individuals homozygous for PWK, and the heterozygous PWK carriers do not have low EXOSC7 abundance (dotted red box). (E) Comparison of the modeled haplotype effects at the *Exosc7* locus reveal a marked lower PWK effect specific to the CC, which is consistent with a recessive effect. Standard error bars are shown. The identity line is included for reference. (F) Mice from the PWK strain with low EXOSC7 abundance were observed, though the effect is not as distinct as in the CC, possibly due to decreased accuracy due to the absence of the bridge sample. Founder strain mice are summarized with mean $\pm$ 2 standard deviation bars.

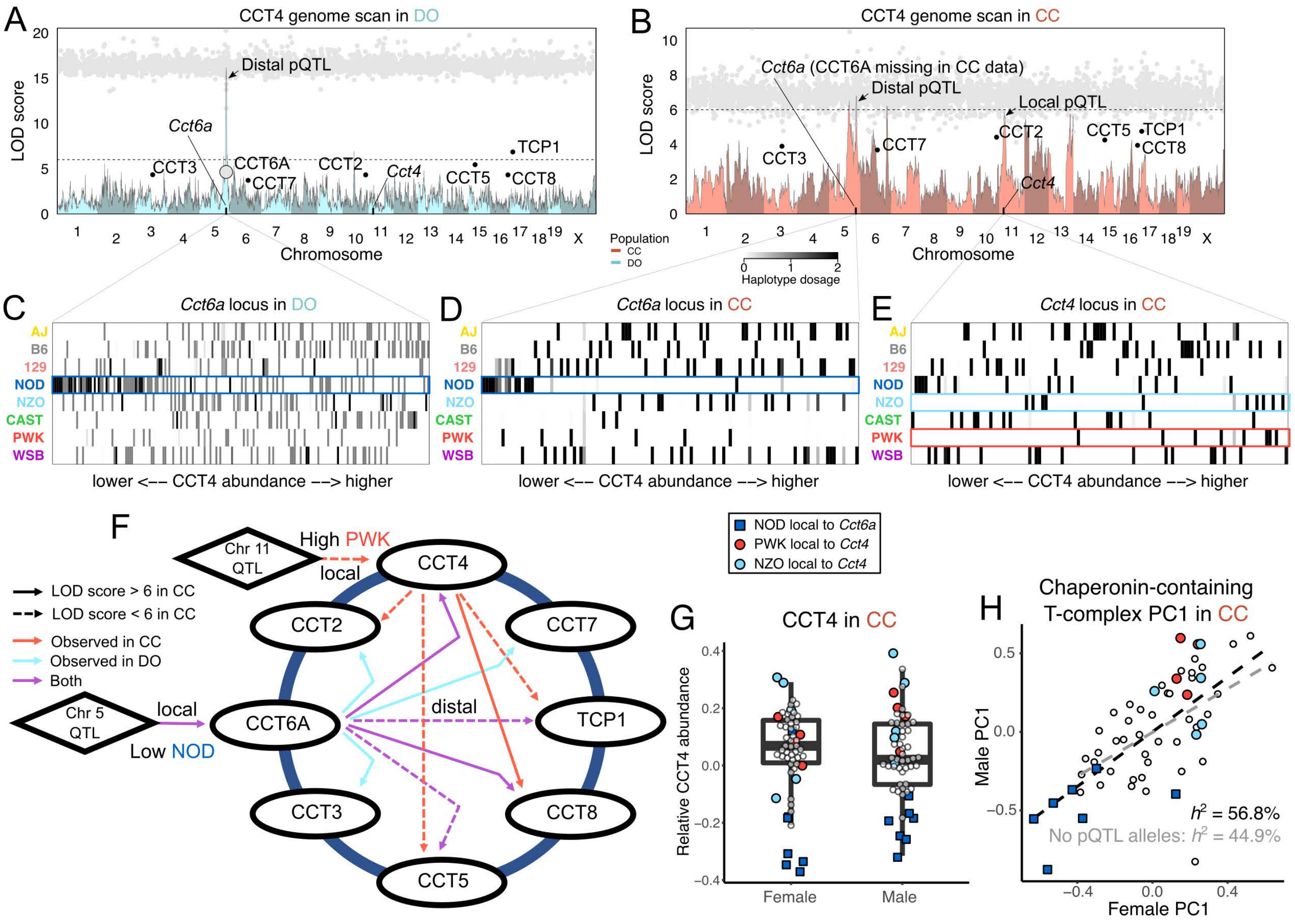

Figure S6. Stochiometry-driven genetic effects on the chaperonin-containing T-complex (CCT-complex), related to Figures 3 and 4. The genome scans for CCT4 overlayed with mediation conditional LOD scores (gray points) for the distal pQTL on chromosome 5 are shown for the (A) DO and (B) CC. Horizontal line at LOD score of 6 included as reference point across genome scans. Founder haplotypes at the *Cct6a* locus are shown for individual mice as heatmaps for the (C) DO and (D) CC. Grey bars indicate heterozygous mice. Mice are ordered along the x-axis according to the abundance of CCT4. The NOD haplotype and its low effect are highlighted with dark blue boxes. (E) Founder haplotypes at the *Cct4* locus for individual CC mice. NZO and PWK haplotypes and their high effects are highlighted with light blue and red boxes, respectively. (F) Mediation analysis for distal pQTL of CCT-complex members reveals population-specific effects for DO (blue) and CC (red) as well as shared (purple). Dashed arrows represent relationships that are based on suggestive LOD score < 6 in the CC. (G) CCT4 abundance is shown for female and male CC mice. Dark blue squares indicate the six strains that have the NOD haplotype at *Cct6a*. Light blue and red circles indicate the strains with NZO and PWK haplotypes at *Cct4*. (H) The CCT-complex PC1 plotted as males vs. females. The black dashed line represents the best fit line based on all strains, and the gray dashed line is the best fit line with strains with the NOD haplotype at Cct6a and NZO and PWK haplotypes at Cct4 excluded.

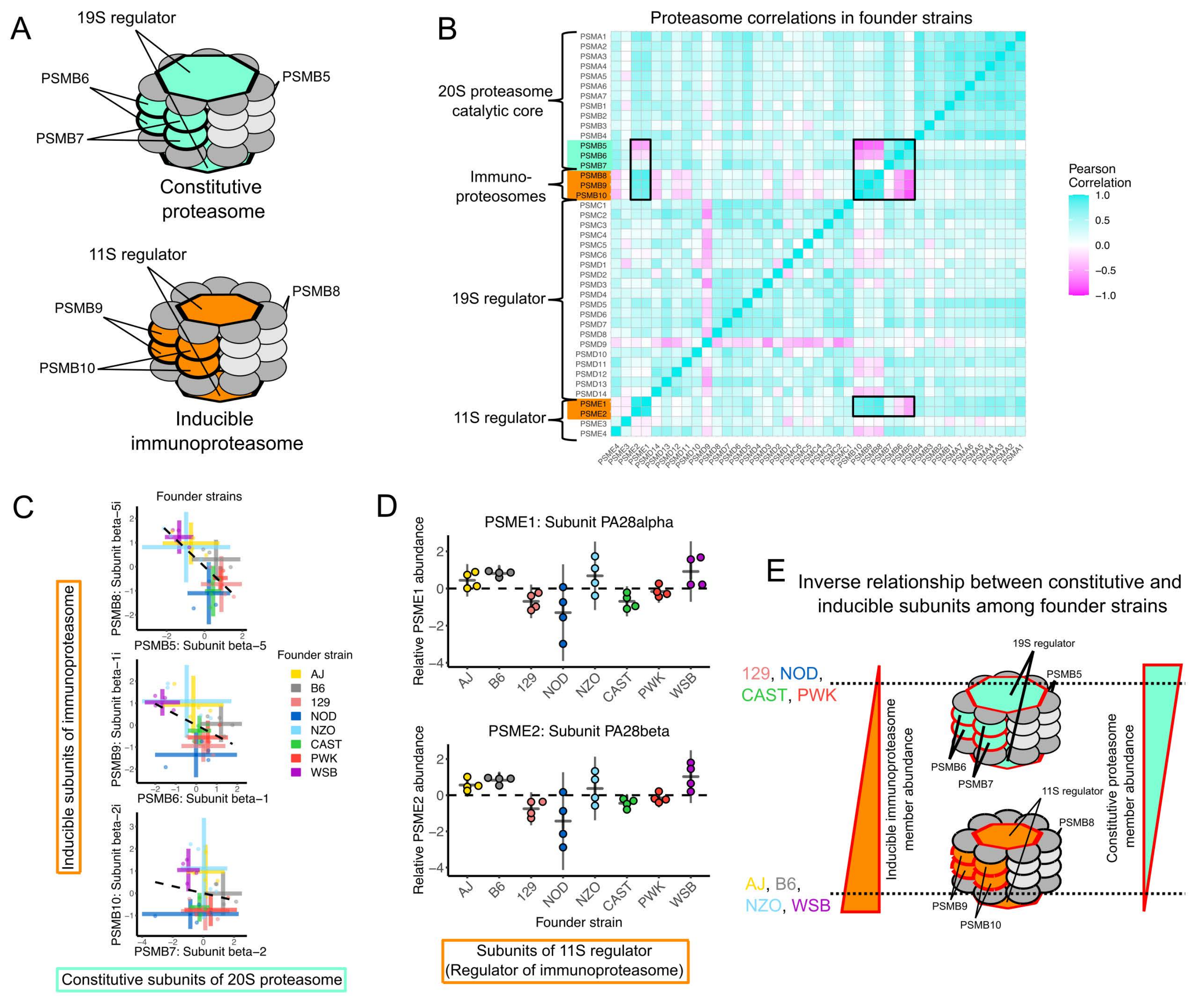

Figure S7. Genetic control of the constitutive and inducible forms of the 26S proteasome, related to Figures 3 and 5. (A) The 26S proteasome is composed of multiple subcomplexes: the 20S proteasome catalytic core (PSMA and PSMB proteins) and 19S regulator (PSMC and PSMD proteins) for the constitutive form, and the inducible immunoproteasomes (PSMB8, PSMB9, and PSMB10) with their 11S regulator (PSME proteins). (B) The correlation of the 26S proteosome proteins in the founder strains of the CC and DO. Black boxes added to highlight correlations between the constitutive and inducible components. (C) Abundances of inducible subunits plotted against their constitutive analogue for the founder strains: (top) PSMB8 vs. PSMB5, (middle) PSMB9 vs. PSMB6, and (bottom) PSMB10 vs. PSMB7. Points and bars are colored by founder strain. (D) Abundances for (top) PSME1 and (bottom) PSME2 in the founder strains. Mean $\pm$ 2 standard deviation bars included. Horizontal line at 0 included for reference. (E) The AJ, B6, NZO, and WSB strains generally have greater abundance of the immunoproteasome subunits compared to the constitutive subunits.

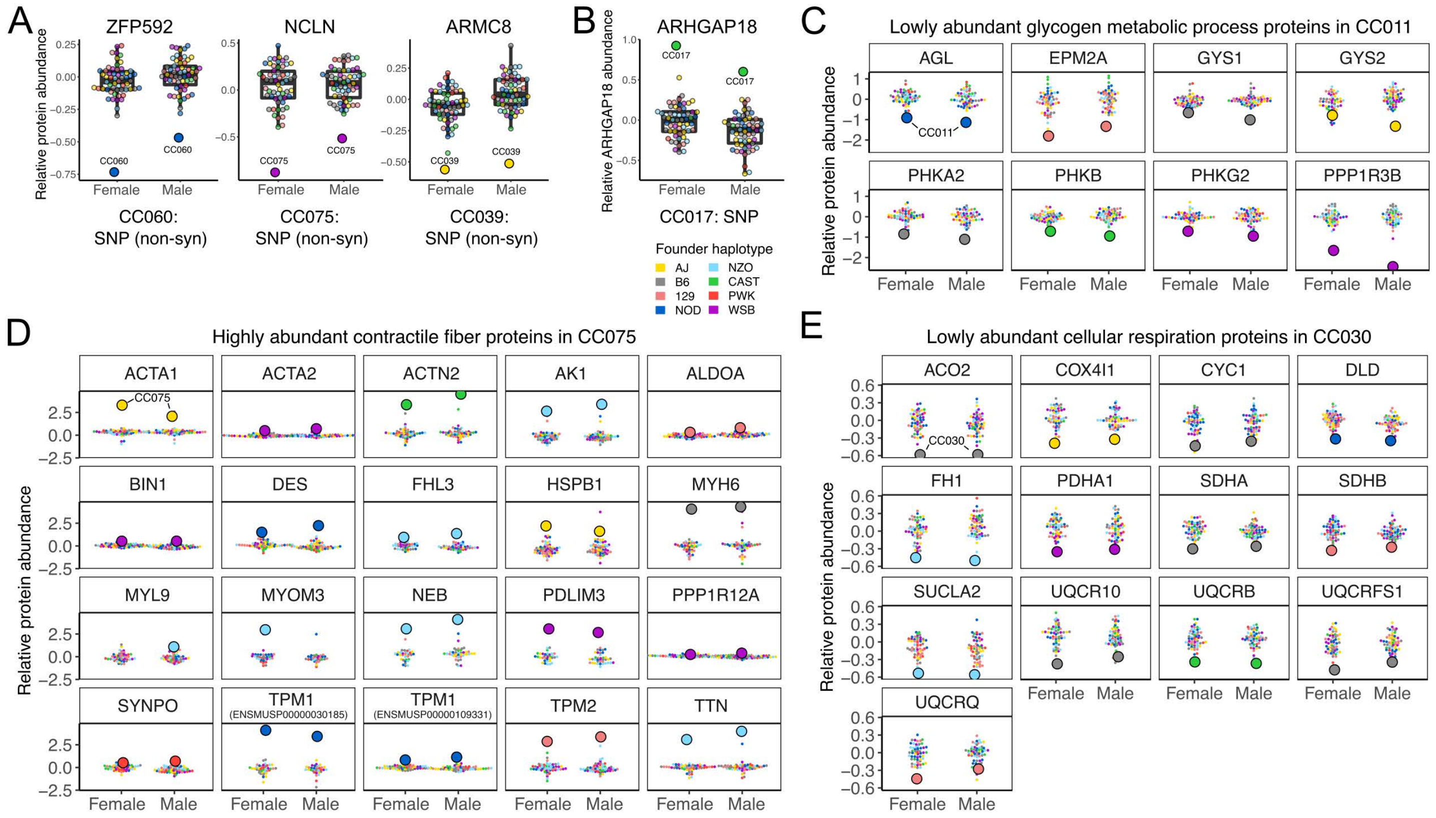

Figure S8. Examples of outlier strains explained by strain-private genetic variants and shared functional pathways, related to Figure 7. (A) Abundances for (left) ZFP592, (middle) NCLN, and (right) ARMC8 in female and male CC mice with outlier strains highlighted. All three strains possess strain-private non-synonymous SNPs in the coding gene, and thus likely represent false low abundance signals due to the reference allele-specific mass-spec quantification. Strain-private variants associated with higher abundance were also observed, such as for (B) ARHGAP18 abundance in CC017, which possessed a strain-private SNP allele. CC strain-specific protein outlier dynamics include (C) low abundance of glycogen metabolic process proteins for CC0011, (F) high abundance of contractile fiber proteins for CC075, and (E) low abundance of cellular respiration proteins for CC030. Point color corresponds to the founder haplotype at the gene locus of the specified protein. See also Table S7.
